# Scatter Removal in Photon-Counting Dual-Energy Chest X-Ray Imaging Using a Moving Block Method: A Simulation Phantom Study

**DOI:** 10.3390/s25216734

**Published:** 2025-11-03

**Authors:** Bahaa Ghammraoui, Yee Lam Elim Thompson

**Affiliations:** Division of Imaging, Diagnostics, and Software Reliability, Office of Science and Engineering Laboratories, Center for Devices and Radiological Health, U.S. Food and Drug Administration, Silver Spring, MD 20993, USA

**Keywords:** photon counting, dual-energy chest radiography, scatter correction, moving blocker, Monte Carlo, DEC, DSE

## Abstract

This work investigates the impact of scatter correction on photon-counting dual-energy chest radiography using a moving block method, focusing on quantifying improvements with the IEC 62220-2-1 dual-energy metrics. A modified LucAl-based chest phantom with PMMA and aluminum inserts was modeled in three sizes (small, standard, large) to represent different patient sizes. Monte Carlo simulations with MC-GPU and the Photon Counting Toolkit were used to simulate a CdTe photon-counting detector with two energy thresholds at 30 and 70 keV. Scatter was estimated from blocker shadows at 25 positions, interpolated across the field of view, and smoothed with a Gaussian filter (σ=5.0 mm), then subtracted separately from low- and high-energy images. Performance was evaluated using the per-feature dual-energy contrast (DEC) and the kerma-normalized dual-energy subtraction efficiency (DSE) with all acquisitions normalized to an entrance air kerma of 1 mGy to reflect clinical exposure conditions. In simulations, the moving block estimate reproduced the true scatter distribution with an average pixel-wise error of 0.4%. Scatter contamination introduced visible artifacts in the dual-energy subtraction images, particularly in aluminum-enhanced (Al-enhanced) images, and reduced contrast for target materials by up to 25%, as reflected in both DEC and DSE values at a fixed dose. Scatter correction restored image contrast, increased DEC for target materials while keeping non-target DEC low, and reduced edge artifacts across phantom sizes with the largest gains in the large phantom. These results support the moving block method as a dose-neutral strategy to improve dual-energy subtraction performance in photon-counting chest radiography.

## 1. Introduction

Dual-energy chest radiography (DECR) is an established imaging technique that enhances visualization of thoracic structures by leveraging the energy-dependent attenuation properties of X-rays. By acquiring images at high and low energy levels, DECR enables suppression of unwanted structures, such as bones, thereby enhancing the visibility of soft tissue when diagnosing lung pathology [1,2]. Recent advances, particularly in photon-counting X-ray detectors (PCDs), offer improved energy separation, higher spatial resolution, the ability to operate with zero electronic noise, and simultaneous acquisition of low- and high-energy images, which can reduce radiation dose and minimize motion artifacts, making them well-suited for dual-energy imaging [3]. However, one of the key challenges in DECR remains scatter contamination, which can degrade image contrast and reduce the accuracy of dual-energy subtraction.

Several strategies have been explored for scatter mitigation in dual-energy radiography. Hardware methods such as anti-scatter grids or larger air gaps lower the scatter-to-primary ratio but leave residual, energy-dependent scatter that can bias the low- and high-energy images differently [4]. Slot scanning or scanned-beam systems reduce scatter geometrically but require specialized motion and hardware [5]. Algorithmic approaches estimate scatter from prior models or convolution kernels tuned to thickness or path-length maps; these methods can work with single-shot acquisitions but require calibration and the kernel is energy-dependent [6,7]. Learning-based estimators have also been proposed, but performance can vary outside training conditions and across energy bins [8]. Acquisition-based sampling methods, such as beam-stop arrays and moving blockers, measure the scatter field directly at the detector by locally occluding the primary signal, enabling per-bin sampling and correction while the patient geometry remains unchanged [9,10,11]. Within this family, the moving block method trades a short sequence of low-dose frames for dense spatial sampling of the scatter field. In photon-counting systems it is practical because frames can be acquired without an electronic noise penalty and the total kerma is shared across frames. It provides a data-driven scatter estimate in each energy bin with minimal calibration and avoids fixed shadow artifacts associated with static beam-stop arrays.

This study focuses on scatter correction in photon-counting dual-energy chest radiography and highlights its impact on image quality through simulation. We evaluate performance using the IEC 62220-2-1 [12] dual-energy metrics: the per-feature dual-energy contrast (DEC) and the kerma-normalized dual-energy subtraction efficiency (DSE). Addressing scatter is particularly important for photon-counting systems because their energy sensitivity makes them more affected by scatter. Scattered photons, predominantly at lower energies, can be misclassified into incorrect energy bins, which reduces the accuracy of dual-energy subtraction and affects imaging reliability. This spectral distortion degrades image contrast, so effective scatter correction is essential for optimizing dual-energy imaging. Moreover, since PCDs can acquire multiple frames without electronic noise penalties, they enable advanced acquisition-based scatter correction techniques, such as the moving block method [13].

A key aspect of this research is the use of a simplified chest phantom model, adapted from established phantom designs, to provide a controlled and adaptable framework for assessing DECR performance. While anthropomorphic phantoms offer realistic anatomy, their complexity and cost limit their practicality for parametric scatter studies [14]. The simplified phantom design used here offers a cost-effective and flexible alternative, allowing systematic evaluation of scatter correction techniques and dual-energy subtraction performance.

This work builds upon prior studies, including the IEC 62220-2-1 standard for dual-energy efficiency assessment. By integrating a moving-block-based scatter correction method into the simulated acquisition process, we demonstrate the potential benefits of scatter removal in DECR. The proposed methodology refines the assessment using DEC and the corresponding DSE sets and underscores the role of scatter correction in improving contrast and image quality in photon-counting dual-energy chest radiography.

### 1.1. Phantom Design

A chest phantom model similar to the LucAl phantom (Standard Dosimetric/Calibration Phantom; Center for Devices and Radiological Health, FDA) was implemented in simulation, following the description by Conway et al. [15,16] and incorporating the inserts proposed in the IEC 62220-2-1 standard [12] with modifications.

**Figure 1:**
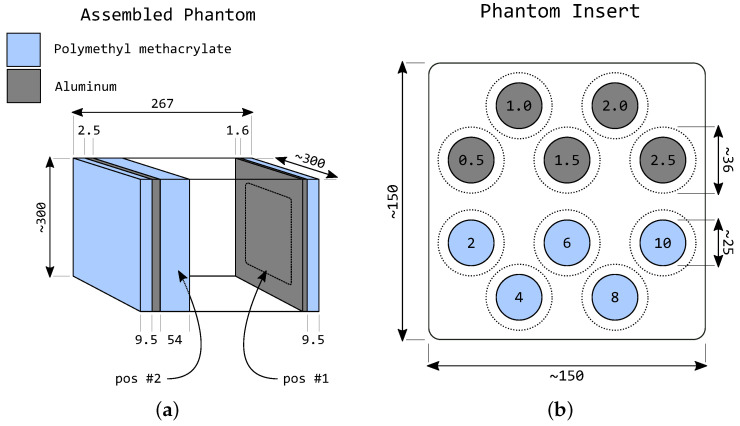
Schematic representation of the modeled LucAl-based chest phantom used in simulations. All dimensions are in millimeters. (**a**) Simulated phantom geometry for the average chest size configuration; (**b**) Simulated arrangement of PMMA and aluminum cylindrical inserts.

The modeled phantom background consisted of 300 mm × 300 mm polymethyl methacrylate (PMMA) plates and an aluminum (Al) layer stack totaling 4.1 mm, as shown in Figure 1a for the standard phantom size. Three total background thicknesses were modeled: 247, 267, and 287 mm, corresponding to small, standard, and large configurations, respectively. These totals were achieved by combining 4.1 mm Al with PMMA thicknesses of 53, 73, and 93 mm and a 190 mm air gap. These thicknesses and the air gap were selected to approximate primary transmission and scatter conditions of lung-field regions at 120 kVp for small, standard, and large body habitus, consistent with the design intent of IEC 62220-2-1.

The LucAl phantom has been reported to exhibit good spectral equivalence to Alderson-Rando male and female anthropomorphic phantoms, supporting its use for simulating realistic imaging conditions [16,17]. In this work, additional phantom sizes were modeled to represent variations observed in different patient body habitus.

The insert configuration included 10 cylindrical volumes of PMMA and Al (five of each), each with a diameter of 25 mm. PMMA inserts had thicknesses of 2 mm, 4 mm, 6 mm, 8 mm, and 10 mm, while the Al inserts had thicknesses of 0.5 mm, 1.0 mm, 1.5 mm, 2.0 mm, and 2.5 mm, as illustrated in Figure 1b.

### 1.2. Simulation of Dual-Energy Chest Radiography with Photon-Counting Detectors

Ideal monoenergetic images with 1 keV energy spacing from 5 keV to 100 keV were generated using the MC-GPU Monte Carlo simulation tool, assuming an ideal photon-counting detector (PCD) model with 100% quantum efficiency and ideal energy response. Both primary (direct) and scattered photons were simulated. To model a realistic detector response, the MC-GPU output was processed using the publicly available Photon Counting Toolkit (PcTK) [18,19], which incorporates spectral distortion and charge-sharing effects in CdTe detectors. The combined MC-GPU_v1.3 and PcTK processing code is available through the DIDSR GitHub repository https://github.com/DIDSR/MCGPUv1.3_PCD, accessed on 1 October 2024.

The simulated imaging system employed a tungsten anode X-ray source operated at 120 kVp with 4 mm aluminum filtration. The source-to-detector distance (SDD) was 180 cm, and the source-to-isocenter distance (SID) was 150 cm. The detector size was 40×40 cm^2^ with a native pixel pitch of 100 μm and thickness of 750 μm. Detector binning was applied in a 10 × 10 pixel configuration, yielding an effective pixel size of 1 mm. The incident photon spectrum and fluence were determined following the exposure guidelines of Report 78 [20], which provides the X-ray spectrum in terms of the number of incident photons. The total number of simulated photons was scaled to correspond to an air kerma of 1 mGy at the detector entrance, ensuring dose normalization.

The detector simulation parameters were as follows: CdTe density of $5.85{g/\mathrm{cm}}^{3}$; charge cloud radius r=8 μm; electronic noise standard deviation σ=1.6 keV; and two energy thresholds: low-energy (LE) at 30 keV and high-energy (HE) at 70 keV.

For scatter correction, 25 frames were simulated to mimic the moving block method, with a tungsten rod positioned at 25 uniformly spaced locations along the detector plane. Each frame was simulated with an exposure corresponding to one twenty-fifth of the total dose (1 mGy/25), so that the combined dataset was equivalent to an overall air kerma of 1 mGy at the detector entrance. The rod’s attenuation profile was calculated via ray tracing and applied exclusively to the primary photon signal, ensuring that the simulated scatter distribution remained unchanged. The tungsten rod had a physical width of 1 mm and a thickness of 2 mm along the source-to-detector axis. It was positioned 20 cm from the source (magnification at the detector plane M≈9). To span the 40 cm detector width, the rod was translated laterally by 4.44 cm at the blocker plane using 25 uniformly spaced positions (blocker plane step ≈1.85 mm; projected step at the detector ≈1.67 cm).

## 2. Dual-Energy Subtraction Method

Dual-energy subtraction was performed on the simulated low- and high-energy images after applying an open-beam correction to both datasets. The open-beam correction was implemented using a logarithmic transformation with a reference image generated from a simulated 60 mm PMMA slab. The corrected images were then combined using a weighting factor *w* chosen to minimize the contribution of the undesired material in each subtraction image.

The weighting factors were determined from the simulated response of a 1.5 mm thick aluminum insert and a 6 mm thick PMMA insert. Specifically, for the PMMA-enhanced (soft tissue) image, $w_{\mathrm{PMMA}}$ was selected to cancel the 1.5 mm aluminum insert signal. Conversely, for the Al-enhanced (bone) image, $w_{\mathrm{Al}}$ was selected to cancel the 6 mm PMMA insert signal. The dual-energy subtraction images were then computed as follows:

(1) $$I_{\mathrm{PMMA}}=I_{L}-w_{\mathrm{PMMA}}I_{H},$$

(2) $$I_{\mathrm{Al}}=I_{H}-w_{\mathrm{Al}}I_{L},$$

where $I_{L}$ and $I_{H}$ are the logarithmically corrected low- and high-energy images, respectively. For consistency with the evaluation notation used later, we denote the PMMA-enhanced image by $I_{s}\equiv I_{\mathrm{PMMA}}$ and the Al-enhanced image by $I_{b}\equiv I_{\mathrm{Al}}$.

### Scatter Estimation and Correction

Scatter estimation was performed by analyzing the shadow regions created by the simulated moving block method. To minimize contamination from transmitted photons, only the central pixels within each rod shadow were used for scatter measurement. Since scatter values were available only at discrete rod positions, cubic interpolation was applied to reconstruct a continuous scatter distribution across the detector. To further reduce noise and suppress interpolation artifacts, Gaussian filtering with σ=5.0 mm was applied, chosen slightly below the projected blocker shadow width (∼9 mm) to suppress interpolation noise without oversmoothing the slowly varying scatter field. Scatter correction was performed independently for the low-energy and high-energy images. The interpolated and smoothed scatter estimates were subtracted from their corresponding images, yielding scatter-corrected datasets for both energy bins.

## 3. Evaluation of Dual-Energy Subtraction Performance

We evaluated performance following IEC 62220-2-1 [12], reporting the per-feature dual-energy contrast (DEC) and the kerma-normalized dual-energy subtraction efficiency (DSE). Let $I_{s}$ denote the PMMA-enhanced (soft tissue) image and $I_{b}$ the Al-enhanced (bone) image.

For each insert i∈{1,…,5} (per material) and for two independent realizations (1) and (2) of the same tissue-subtracted image type, DEC is

(3) $${\mathrm{DEC}}_{t}^{M}\left(i\right)=\frac{\left({\bar{I}}_{F_{i,t}}^{\left(1\right)}-{\bar{I}}_{B_{i,t}}^{\left(1\right)}\right)+\left({\bar{I}}_{F_{i,t}}^{\left(2\right)}-{\bar{I}}_{B_{i,t}}^{\left(2\right)}\right)}{\sqrt{2}\sqrt{\mathrm{var}\left(I_{F_{i,t}}^{\left(1\right)}\right)+\mathrm{var}\left(I_{B_{i,t}}^{\left(1\right)}\right)+\mathrm{var}\left(I_{F_{i,t}}^{\left(2\right)}\right)+\mathrm{var}\left(I_{B_{i,t}}^{\left(2\right)}\right)}},$$

where t∈{s,b} indicates the image type (PMMA-enhanced or Al-enhanced), M∈{PMMA,Al} indicates the feature material measured in that image, overbars denote ROI means, and var(·) denotes ROI variances. DEC is dimensionless.

The DSE for each tissue-subtracted image is the set of its two kerma-normalized DEC curves:

(4) $${\mathrm{DSE}}_{s}=\left\{\frac{{\mathrm{DEC}}_{s}^{\mathrm{PMMA}}}{\sqrt{K_{a}}},\frac{{\mathrm{DEC}}_{s}^{\mathrm{Al}}}{\sqrt{K_{a}}}\right\},\;\;{\mathrm{DSE}}_{b}=\left\{\frac{{\mathrm{DEC}}_{b}^{\mathrm{PMMA}}}{\sqrt{K_{a}}},\frac{{\mathrm{DEC}}_{b}^{\mathrm{Al}}}{\sqrt{K_{a}}}\right\},$$

with units of mGy^−1/2^. In our simulations, the entrance air kerma was fixed at $K_{a}=1$ mGy; therefore numerical values of DSE and DEC coincide (i.e., $\mathrm{DSE}=\mathrm{DEC}/\sqrt{K_{a}}$).

For clarity, we report ${\mathrm{DEC}}_{s}^{\mathrm{PMMA}}$ and ${\mathrm{DEC}}_{s}^{\mathrm{Al}}$ for $I_{s}$, and ${\mathrm{DEC}}_{b}^{\mathrm{PMMA}}$ and ${\mathrm{DEC}}_{b}^{\mathrm{Al}}$ for $I_{b}$; the associated DSE sets are ${\mathrm{DSE}}_{s}=\left\{{\mathrm{DEC}}_{s}^{\mathrm{PMMA}}/\sqrt{K_{a}},{\mathrm{DEC}}_{s}^{\mathrm{Al}}/\sqrt{K_{a}}\right\}$ and ${\mathrm{DSE}}_{b}=\left\{{\mathrm{DEC}}_{b}^{\mathrm{PMMA}}/\sqrt{K_{a}},{\mathrm{DEC}}_{b}^{\mathrm{Al}}/\sqrt{K_{a}}\right\}$.

## 4. Results

### 4.1. Simulation Results for Scatter Estimation and Correction

**Figure 2:**
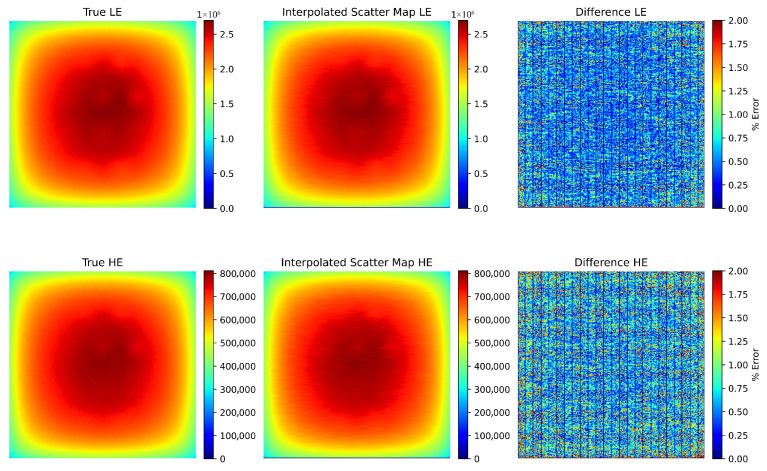
Comparison of true and estimated scatter maps for the standard simulated phantom size. The last column shows the percentage difference, with most pixels exhibiting less than 1% error.

To validate the accuracy of the scatter estimation method, we compared the true scatter map obtained from Monte Carlo simulations with the estimated scatter map using the simulated moving block technique. Figure 2 presents an example comparison for the standard phantom size. The difference map shows that the error is below 1% for all pixels, with an average error of 0.4%.

**Figure 3:**
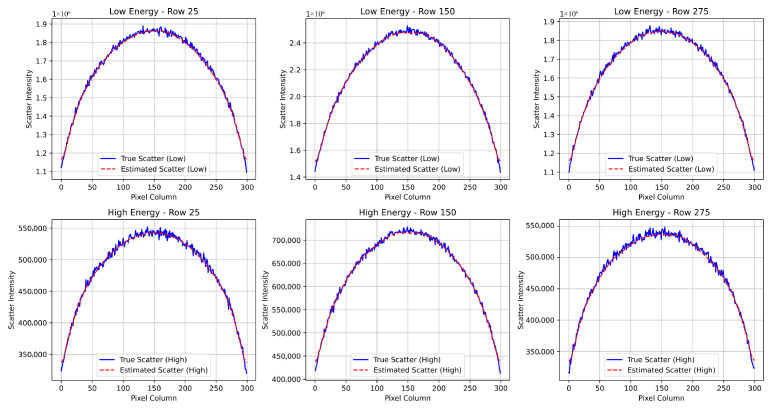
Comparison of 1D scatter profiles at different rows in the simulated scatter maps. The estimated scatter closely matches the true scatter, with the true profiles exhibiting slightly more noise.

To further assess the accuracy of scatter estimation, we extracted 1D profiles at different rows in the low-energy (LE) and high-energy (HE) simulated scatter maps. Figure 3 demonstrates excellent agreement between the estimated and true profiles, with minor discrepancies attributed to the noise reduction applied to the estimated scatter maps.

### 4.2. Impact of Scatter on Dual-Energy Subtraction Images

**Figure 4:**
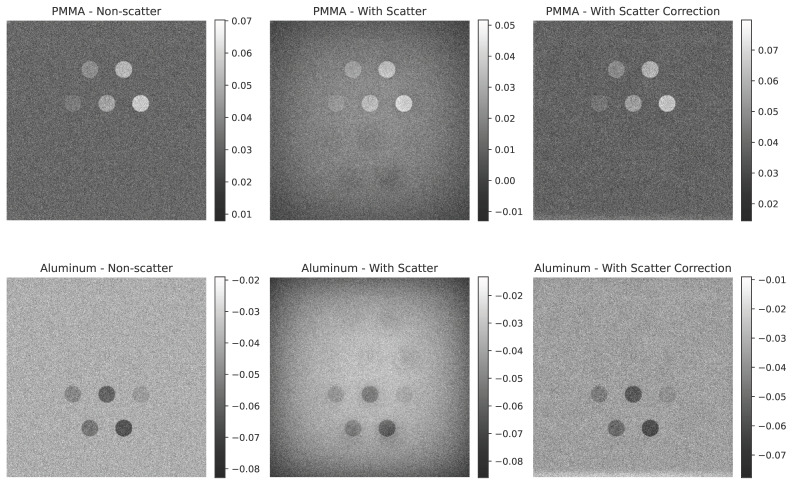
Simulated dual-energy subtraction images for PMMA (**top**) and Al (**bottom**) inserts in the standard phantom size under three conditions: (**a**) without scatter, (**b**) with scatter, and (**c**) after scatter correction. Scatter-induced artifacts are evident in the second row, especially in the Al-enhanced images, but are effectively removed after correction.

**Figure 5:**
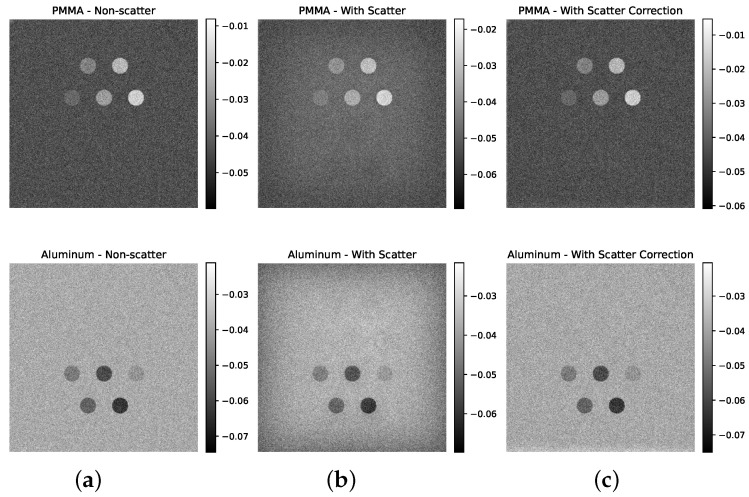
Simulated dual-energy subtraction images for PMMA (**top**) and Al (**bottom**) inserts in the small phantom size under three conditions: (**a**) without scatter, (**b**) with scatter, and (**c**) after scatter correction. Scatter-induced artifacts are evident in the second row, especially in the Al-enhanced images, but are effectively removed after correction.

**Figure 6:**
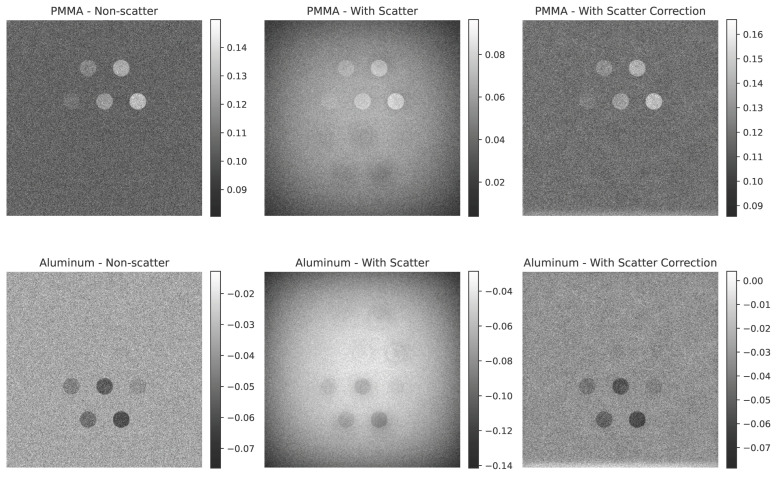
Simulated dual-energy subtraction images for PMMA (**top**) and Al (**bottom**) inserts in the large phantom size under three conditions: (**a**) without scatter, (**b**) with scatter, and (**c**) after scatter correction. Scatter-induced artifacts are evident in the second row, especially in the Al-enhanced images, but are effectively removed after correction.

Figure 4, Figure 5 and Figure 6 illustrate the impact of scatter on the simulated dual-energy subtraction images for the standard, small, and large phantom sizes, respectively. In the absence of scatter (top row), the soft tissue (PMMA-enhanced) and bone (Al-enhanced) subtraction images exhibit ideal material separation. In the presence of scatter (middle row), artifacts appear at the edges of the inserts, particularly in the Al-enhanced images. After applying scatter correction (bottom row), these artifacts are significantly reduced, restoring the desired contrast. For visualization, grayscale intensity ranges were scaled between the minimum and maximum values of each image to maximize contrast, as the dual-energy subtraction images are qualitative and not intended for quantitative comparison.

### 4.3. Quantitative Evaluation Using DEC and DSE

**Figure 7:**
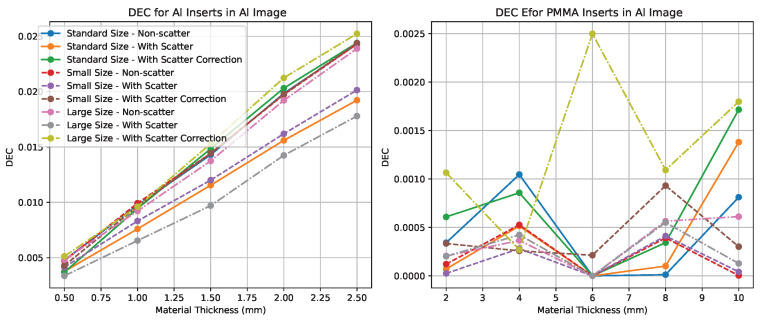
Dual-energy contrast (DEC) measured in the Al-enhanced image, i.e., $\left({\mathrm{DEC}}_{b}^{\mathrm{PMMA}},{\mathrm{DEC}}_{b}^{\mathrm{Al}}\right)$. These two curves form the DSE*_b_* set when normalized by $\sqrt{K_{a}}$. With $K_{a}=1$ mGy, the plotted DEC values are numerically equal to DSE. Scatter correction increases DEC for the targeted material while keeping the non-target DEC low.

**Figure 8:**
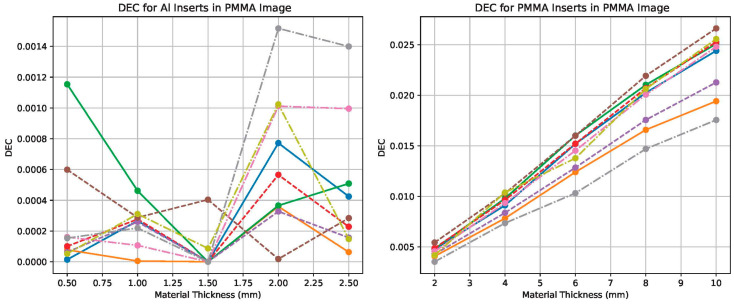
Dual-energy contrast (DEC) measured in the PMMA-enhanced image, i.e., $\left({\mathrm{DEC}}_{s}^{\mathrm{PMMA}},{\mathrm{DEC}}_{s}^{\mathrm{Al}}\right)$. These two curves form the DSE*_s_* set when normalized by $\sqrt{K_{a}}$. With $K_{a}=1$ mGy, the plotted DEC values are numerically equal to DSE. Scatter correction increases DEC for the targeted material while keeping the non-target DEC low.

We computed the dual-energy contrast (DEC) for each feature material in each tissue-subtracted image. With $K_{a}=1$ mGy, these DEC curves are numerically equal to the corresponding DSE sets. For the Al-enhanced image (Figure 7), the DEC for Al inserts increases approximately linearly with insert thickness and improves by up to 25% after scatter correction. For the PMMA-enhanced image (Figure 8), the DEC for PMMA inserts shows the same trend and magnitude of improvement.

Non-target DEC values remain low across all conditions (PMMA in the Al-enhanced image and Al in the PMMA-enhanced image), indicating effective suppression of the undesired material. Small residual artifacts are visible in the large phantom case; these are consistent with the higher scatter fraction and suggest that DEC may not fully capture structured residuals at edges.

Across phantom sizes, the DEC curves agree closely, with larger absolute gains in the large phantom where scatter is highest. These results support the need for scatter correction in photon-counting dual-energy chest radiography and show that the moving block approach increases target-material DEC (and therefore the corresponding DSE sets at the same kerma) while maintaining low non-target DEC.

## 5. Discussion

This work used simulations with a realistic photon-counting detector model to study scatter removal with a moving-block-based method in dual-energy chest radiography. The main finding is that estimating and subtracting a smoothed scatter map from each energy bin restores subtraction image quality and improves DEC for the targeted material across phantom sizes.

There are several limitations. First, the study is simulation based. Although the detector response model (PcTK) includes spectral distortion and charge sharing, other effects (for example, detector nonuniformity, pulse pileup at higher flux, and focal-spot blur) were not varied. Second, the acquisition settings were fixed (120 kVp, two thresholds at 30/70 keV, and 1 mGy entrance kerma); performance may depend on spectrum, threshold placement, and kerma. Third, the blocker sampling used 25 positions with a fixed Gaussian smoothing parameter; different sampling densities or adaptive smoothing could further reduce interpolation bias near high-contrast edges. Finally, motion was not modeled, and while the LucAl-based phantom reproduces lung-field spectral properties, it does not capture the full anatomical complexity of the thorax. Additional validation with anthropomorphic phantoms or patient data is therefore needed to assess clinical generalizability.

Future work will include sensitivity analysis to spectrum and threshold placement, optimization of blocker-position density and smoothing, and experiments with physical phantoms to assess robustness under more realistic conditions.

## 6. Conclusions

This simulation study with a realistic photon-counting detector model investigated scatter effects and a moving-block-based scatter correction for dual-energy chest radiography. Scatter introduced visible artifacts and reduced subtraction contrast, especially near high-attenuation aluminum inserts. Estimating and subtracting a smoothed scatter map from each energy bin restored subtraction image quality and increased the dual-energy contrast (DEC) for the targeted materials by up to 25% while keeping non-target DEC low; at the same air kerma, the corresponding dual-energy subtraction efficiency (DSE) sets improved by the same amount. The approach performed similarly across small, standard, and large phantom sizes.

Future work will examine sensitivity to spectrum and threshold placement, optimize blocker-position density and smoothing, and validate the method on physical phantoms. We will also quantify uncertainty from interpolation and smoothing and assess robustness to detector nonuniformity and motion.

## Data Availability

Data and code are available from the corresponding author upon reasonable request.
